# Developing a Theory of Change model of service user and caregiver involvement in mental health system strengthening in primary health care in rural Ethiopia

**DOI:** 10.1186/s13033-020-00383-6

**Published:** 2020-07-23

**Authors:** Sisay Abayneh, Heidi Lempp, Atalay Alem, Brandon A. Kohrt, Abebaw Fekadu, Charlotte Hanlon

**Affiliations:** 1 Department of Psychiatry, School of Medicine, College of Health Sciences, Addis Ababa University, WHO Collaborating Centre in Mental Health Research and Capacity Building, Addis Ababa, Ethiopia; 2 Faculty of Life Sciences and Medicine, Centre for Rheumatic Diseases, School of Immunology and Microbial Sciences, King’s College London, Weston Education Centre 10, Cutcombe Rd, London, SE5 9RJ UK; 3 Department of Psychiatry, George Washington University, Washington, DC USA; 4 College of Health Sciences, Centre for Innovative Drug Development and Therapeutic Trials for Africa (CDT-Africa), Addis Ababa University, Addis Ababa, Ethiopia; 5 Department of Global Health & Infection, Brighton and Sussex Medical School, Brighton, UK; 6 Centre for Global Mental Health, Institute of Psychiatry, Psychology and Neuroscience, King’s College London, 16 De Crespigny Park, London, SE5 8AF UK

**Keywords:** Theory of Change, Service user involvement, Caregiver involvement, Mental health system, Rural, Ethiopia

## Abstract

**Background:**

The involvement of service users and caregivers is recommended as a strategy to strengthen health systems and scale up quality mental healthcare equitably, particularly in low-and-middle-income countries. Service user and caregiver involvement is complex, and its meaningful implementation seems to be a worldwide challenge. Theory of Change (ToC) has been recommended to guide the development, implementation and evaluation of such complex interventions. This paper aims to describe a ToC model for service user and caregiver involvement in a primary mental health care in rural Ethiopia.

**Methods:**

The ToC was developed in two workshops conducted in (i) Addis Ababa with purposively selected psychiatrists (n = 4) and multidisciplinary researchers (n = 3), and (ii) a rural district in south-central Ethiopia (Sodo), with community stakeholders (n = 24). Information from the workshops (provisional ToC maps, minutes, audio recordings), and inputs from a previous qualitative study were triangulated to develop the detailed ToC map. This ToC map was further refined with written feedback and further consultative meetings with the research team (n = 6) and community stakeholders (n = 35).

**Results:**

The experiential knowledge and professional expertise of ToC participants combined to produce a ToC map that incorporated key components (community, health organisation, service user and caregiver), necessary interventions, preconditions, assumptions and indicators towards the long-term outcomes. The participatory nature of ToC by itself raised awareness of the possibilities for servicer user and caregiver involvement, promoted co-working and stimulated immediate commitments to mobilise support for a grass roots service user organization.

**Conclusions:**

The ToC workshops provided an opportunity to co-produce a ToC for service user and caregiver involvement in mental health system strengthening linked to the planned model for scale-up of mental health care in Ethiopia. The next steps will be to pilot a multi-faceted intervention based on the ToC and link locally generated evidence to published evidence and theories to refine the ToC for broader transferability to other mental health settings.

## Introduction

The involvement of service users and their caregivers at all levels of the mental health system has become a core policy in many countries across the world [1–3]. There is lack of consensus about what precisely definition of service user and caregiver involvement, and there are many terms used often interchangeably (e.g. patient/caregiver engagement/co-production, consumer/family participation, patient and public involvement) in the literature [1, 4, 5]. In this study, service user and caregiver involvement is defined as the active involvement by service users, caregivers and their representatives in decision-making within mental health system in a range of activities including, policy making, planning, service development and delivery, monitoring and evaluation or quality assurance, research, training and education, peer support and case management, and advocacy within the health system starting from their expertise gained from experience [1]. The involvement of service user and caregiver can take place at multiple levels: the *micro*-*level* (e.g. in individual care decision-making, planning and management), *meso*-*level* (e.g. in local service planning, monitoring and evaluation, advocacy, training and recruitment of staff, input into guidelines), and *macro*-*level* (e.g. policy making, national level planning and advocacy) [1, 5, 6].

There is explicit international policy direction from the World Health Organization for national mental health systems to empower and involve service users in mental health system strengthening [7, 8]. The same directive has become a policy imperative and is therefore firmly embedded in policy documents of many high income countries [3, 9].

There is evidence from high income countries of many benefits of service user and caregiver involvement for: (i) the healthcare system (e.g., better access to, and acceptability of health care services) [10, 11]; (ii) for health professionals (e.g., improved attitudes, better understanding of service users’ and caregivers’ needs) [10–13], and (iii) improved knowledge about mental health and available services, and networking among service users and caregivers [10–13].

In low-and-middle income countries (LMICs), service user and caregiver involvement has been widely recommended as an essential ingredient of strengthening weak mental health systems [14, 15], which has potential to increase the likelihood of scale-up of appropriate and quality mental healthcare [16, 17], and reduce the treatment gap for quality care [8, 18]. However, in LMICs, there is less prioritization and government support for either mental healthcare provision or involvement of service users [19, 20]. Furthermore, there are often no policies and laws to direct mental health programs and/or the policies and laws are not fully in line with human rights recommendations (e.g., service user participation) or are poorly implemented [19, 20]. Service users and caregivers are still exposed to stigma and discrimination [21, 22] and have multiple unmet needs [22], including symptoms of illness and disability [23], premature mortality [24, 25], and human rights abuses (e.g., being chained or kept in isolation) [21, 26, 27].

While the importance of service user and caregiver involvement in the mental health system is clear, the question of how to implement participation in practice remains a global challenge. The involvement of services and caregivers is a complex process that (1) has been variously defined [1, 5]; (2) is characterized by multiple and often inequitable interactions at the micro-level, the meso-level, and at the macro-level [1, 6, 28]; and (3) requires resources and actions of actors at multiple levels to build a supportive environment [1, 12, 29]. This complexity can be a barrier to developing consensus in relation to (i) the meaning of involvement, (ii) understanding the goals, (iii) identifying the appropriate model and (iv), the expectations, roles and responsibilities of stakeholders for service user and caregiver involvement [1, 11, 30, 31]. Recommended approaches to complex intervention development and implementation [32] have not been applied to articulate what the complex nature of service user involvement might look like in practice [1, 6, 28].

A growing body of development and health actors recommend Theory of Change (ToC) for dealing with complex health interventions [33–35]. ToC has been adopted in some LMICs, including Ethiopia, to develop, implement and evaluate interventions in mental healthcare [34, 36, 37]. ToC is a participatory approach to explore processes for change; “a theory of how and why an initiative works” [38], which both develops an intervention using the experience and expertise of the participants, and documents key indicators that allow systematic evaluation of processes and outcomes of the intervention (e.g., service user and caregiver involvement) for expected steps on the hypothesized causal pathway to impact [35]. Furthermore, several scholars suggested that ToC gives insights not only into intended change, but also the model of action or practice and unforeseen consequences [39–41].

However, there is little published evidence of the application of ToC to the involvement of service user and caregivers in mental health system strengthening. The main objective of this study was, therefore, to describe our experiences of bringing together service users, caregivers, and other key stakeholders to use a ToC approach to develop a model of how best to involve service user and caregivers in mental health system strengthening in primary healthcare in rural Ethiopia.

## Methods

### Setting

This study was conducted as part of the ‘Emerging mental health systems in low- and middle-income countries’(Emerald) project, which investigated the health system requirements for successful improvement of integrated mental health care in six LMICs(Ethiopia, India, Nepal, Nigeria, South Africa and Uganda) [42, 43]. Emerald built upon the PRogramme for Improving Mental health carE (PRIME), an implementation research project which developed, implemented and evaluated an integrated district mental health care plan in collaboration with local stakeholders [44, 45].

This study was undertaken at both the national level in the capital city, Addis Ababa, and the district level in Sodo district, Gurage Zone, located about 100 km south of Addis Ababa. Sodo district had a population of 161 952 people in 2007 [46]. Around 90% of the district population resides in rural areas and most are reliant on subsistence farming [47]. Most of the population in the district follow Orthodox Christianity and are from the Gurage ethnic group. The official language of the district is Amharic [46]. There is one primary hospital with an outpatient psychiatric service in the main town, and eight health centres, four of which are located within the three towns of the district. The primary hospital and all eight health centres have functioning mental health services, developed and implemented recently as part of PRIME [44, 45]. The primary hospital is staffed by a psychiatric nurse and the health centres have general health workers (health officers, nurses and midwives) trained in mental healthcare according to the World Health Organization’s mental health Gap Action Programme (mhGAP) [48]. Each health centre serves a population of about 25,000–40,000 people [49]. Each sub-district has a health post (lowest statutory healthcare facility), covering 3000–5000 population. The health posts are staffed by a pair of community health workers called health extension workers (HEWs). The HEWs are high school graduates with 1 year of training in prevention and promotion activities [50]. A minority of HEWs have received government training in mental health as part of their upgrading to level IV. PRIME has also provided capacity building training for HEWs, in addition to facility-based workers, and established a multi-sectoral community health advisory board (CAB) [51]. The CAB includes representation from key members of the district leadership (security, gender office, women and youth affairs, religious affairs and education), the community and service users and caregivers, and is chaired by the head of the district health office [52]. The CAB meets twice a year to oversee and advise PRIME and support strengthening of the mental health system [51].

### ToC workshop participants

Two ToC workshops were held in 2017 to engage stakeholders in tackling the challenge of how best to embed service user and caregiver involvement in mental health system strengthening. The first ToC workshop was carried out with seven participants (three male, four female) from Addis Ababa University, Department of Psychiatry, who had extensive experience working to expand mental health care in the study site, as well as experience working with service users and advising the Federal Ministry of Health on policy and service planning. The participants had diverse professional backgrounds (psychiatry, psychology, social work, pharmacology, epidemiology, public health).

The second ToC workshop took place in Sodo, and involved 24 participants selected purposively [53] on the basis of being key stakeholders in mental healthcare or possessing expertise in service planning. The participants included: (i) district level government office representatives(managers of the district health administration, focal person for mental health, women and children’s office, youth and sport office, social affairs office), (ii) community-based organizations and leaders (religious and faith-based leaders, non-governmental organization representatives), (iii) representatives of service providers, service users and caregivers, and (iv) five senior mental health researchers from Addis Ababa University who had also participated in the first ToC workshop to provide cross-learning and link local perspectives with national scale-up plans.

### Procedures

**Fig. 1:**
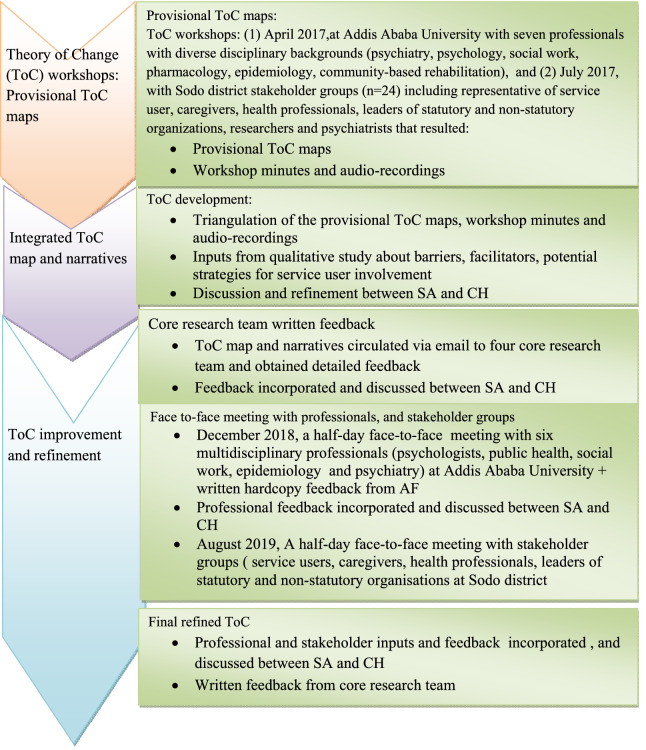
ToC development process

The ToC development underwent five iterative activities in three stages (See Fig. 1 for a schematic depiction of the process).

The first stage involved provisional ToC development using workshops. The first ToC workshop was conducted at Addis Ababa University in April 2017. The second ToC workshop was conducted in Sodo district in July 2017. The ToC was co-facilitated by senior psychiatrists and researchers (AF and CH). Both authors are trained in the use of ToC and had experience of facilitating ToC workshops with diverse stakeholders, including the PRIME project that involved most of the participants who participated in this study. As mental health professionals, both AF and CH were mindful of how they would be perceived by the participants and sought to emphasise that the focus of involvement went beyond narrow biomedical concerns. Power differentials between the facilitators and the Sodo participants were reduced by building on existing collaborative relationships where trust has been build up and participants are comfortable expressing opinions. In both workshops, the facilitators introduced and stated the objectives of the workshop, provided a brief description of the ToC approach and moderated discussions. The processes used to create the ToCs started by defining the desired impact and long–term outcomes, and then worked backwards iteratively to map out preconditions, interventions, assumptions and indicators to generate desired change [33]. The process was assisted by writing down the preconditions, interventions, assumptions, and indicators suggested by the participants on sticky notes on a wall. The two workshops led to two provisional ToC roadmaps.

Second stage, SA drafted the ToC by combining the provisional ToC road maps, minutes of the workshops and inputs from a formative qualitative study detailed elsewhere [54]. In that qualitative study, in-depth interviews were conducted with policy makers/planners, health professionals/facility heads, service users and caregivers from the current study setting and at the national. We found that all groups of participants supported service user and caregiver involvement in mental health system strengthening; however, hardly any respondents had prior experience of service user involvement. Key barriers to involvement identified included limited knowledge of stakeholders about how to work together, service user/caregiver lack of experience and opportunities to be involved, lack of service user networks/associations, and lack of systemic collaboration among stakeholders to support service user involvement. Pervasive stigmatizing attitudes and discrimination operating at multilevel (the health system, the local community and individuals) tended to disqualify service user and caregiver involvement from full social acceptance, marginalize them and hinder their active involvement. All groups of respondents identified a need for awareness-raising and training to equip service users, caregivers, service providers and local community for effective involvement. The findings of the study informed the development of the ToC, particularly to triangulate components of the interventions, barriers, facilitators, and capacity building needs for participation.

The third stage of the ToC development process involved further stakeholder consultation to refine the ToC, comprising: (1) core research team written feedback, (2) consultation meeting with professionals(n = 6, e.g. psychology, social work, public health, psychiatry, epidemiology backgrounds), and (3) Community stakeholder group meeting.

The revised ToC was then discussed and validated in a half-day face-to-face meeting with stakeholder groups including service users (n = 7), caregivers (n = 5), health professionals (n = 8), leaders of statutory and non-statutory organisations (n = 15). The workshop was convened at Sodo district and held in August 2019. After a brief presentation to recap the ToC process and existing ToC map, the group was encouraged to refine and validate the draft ToC against the practical problems encountered in the local district and their knowledge, e.g. of what may work in this context.

### Data collection

The data for this study came mainly from the workshops and meeting minutes, and the provisional ToC maps developed during the workshops. The first ToC workshop was audio-recorded and notes were taken by the first author. These formed the basis for a detailed report of the ToC, which was checked against the audio files for completeness. In the second ToC workshop, minutes were taken. The first (national level) workshop lasted 3:30 h, and the ToC in Sodo district lasted 2:10 h. Data from the formal process documentation of ToC workshops, the two draft ToC maps and consultation meetings were reviewed and combined to develop the final ToC map. We included in the result some illustrative quotes from the minutes checked with first author notes and audio-recordings.

## Results

**Fig. 2:**
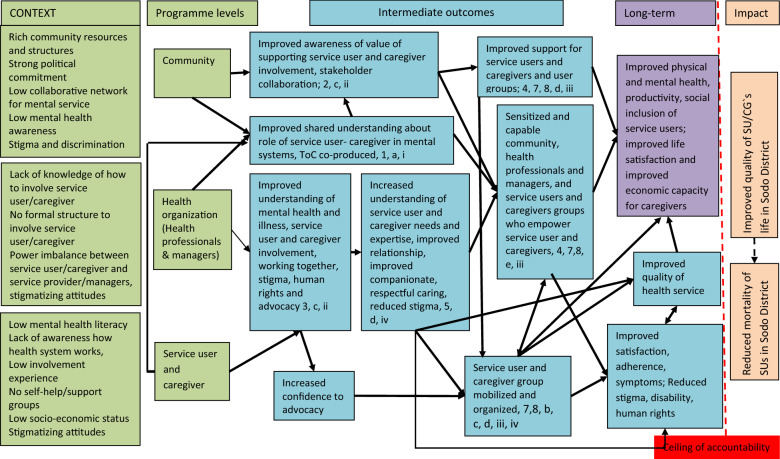
ToC map for developing service user and caregiver involvement in mental health system strengthening in rural Ethiopia. **Example assumptions**: Willingness to (1) involve in ToC workshop, (2) undergo consultative workshop on service user and caregiver mobilization and support, (3) undergo participatory capacity building training(4) form collaborative community (5) involve in participatory action plan development, implementation and evaluating the ToC for service user and caregiver involvement ;(6) Availability of reliable medication supply; (7) CAB’s willingness to mobilize resources to service user and caregiver; (8) Non-governmental organization support to service user and caregiver. **Example indicators:** i. Number of people participated in ToC workshop, consultative workshop, capacity building training and involvement plan. ii. Changes in knowledge and perception pre and post training, experiences gained developing and evaluating the plan for service user and caregiver involvement. iii. Service user and caregiver groups established, and support generated. iv. Experience of involvement in participatory action plan development, implementation and evaluation. **Example interventions** a. Conduct (a) ToC workshop, (b) consultative workshop (c) capacity building participatory training, (d) facilitate participatory involvement in mental health system, (e) Assist service user and caregiver and their group

The details of the ToC workshop participants are presented in Table 1. The following sections will describe details of (i) the finally agreed ToC map (Fig. 2) and (ii) narratives of the process of ToC development to highlight the programme levels, preconditions, assumptions [1–8], indicators (i–iv), and interventions (a–e).

**Table 1:** Theory of Change workshop participants

Stakeholder group	N	Female	Literate
ToC 1—National			
Psychiatrists	4	2	4
Researchers	3	2	3
ToC 2—Sodo district			
Service users	1	1	1
Caregivers	1	0	1
Local government administrators	5	2	5
Health workers	4	1	4
Community representatives	4	0	4
Senior psychiatrists and researchers	9	4	9

### Consensus on outcomes and impact

In the first ToC workshop, the participants suggested (i) improved quality of mental healthcare as the long-term outcome and (ii) better quality of life for service users and caregivers as the ultimate impact of the study. In contrast, members of the second ToC workshop advocated for enhanced (i) physical and mental health, (ii) work productivity, (iii) social inclusion for service users (e.g. Community activities);(iv) improved life satisfaction and (v) economic capacity for caregivers as long-term outcomes. Furthermore, reduced (vi) homelessness and (vii) mortality of service users was identified as a desired impact that went beyond the ceiling of accountability of the project. During the follow-up consultation meetings, the participants came to a consensus on the long-term outcomes, leading to minor modifications to the impact (improved quality of life and reduced mortality of service users in the district). During meetings with health professionals and researchers, concern was expressed about the feasibility of some aspects of the long-term outcome (e.g., work productivity and economic capacity). This concern was presented to stakeholder groups at the meeting at Sodo district and stimulated a heated discussion, with the group expressing their firm belief in the necessity and feasibility of the outcomes. Some participants justified the ambitious long-term outcomes, recounting that they, at the beginning of the PRIME project, had been pessimistic about the viability of integration of mental health care within primary care in the district, but that their perspectives had changed in a very relatively short time after seeing what could be achieved. For example, one participant from the health professional stakeholder group stated:

*“When PRIME project conducted its project sensitization workshop, I and many of my acquaintances who attended that meeting considered many of the project objectives as impossible dreams and mere wishes….but now in a very short time all became true…we have mental health service integrated at primary healthcare delivered with professionals working there, which reduced enormous problems families used to struggled with… maybe this is the best experience that others can take … now we have many experiences….no doubt if we collaboratively work and tap our rich social capital of supporting each another and effectively use our community resources….. we can achieve.” *(Health professional at Sodo district)

The positive changes observed in service users with severe mental health conditions after accessing the new service, now productively working, attending school, farming, and carrying out their daily home routines, were given as evidence of the achievability of the outcomes. In addition, the district government commitment and action to cover costs of medication, combined with an emerging government focus on community-based care and economic empowerment of people under difficult circumstances were considered conducive to success.

The participants identified the necessary interventions and preconditions to be in place to achieve the outcomes (See Table 2).

**Table 2:** Summary of level of intervention, key tasks, intervention and preconditions

Levels of intervention	Key tasks	Intervention	Preconditions
Local community level	Mobilize resources Raise awareness about mental illness, roles or service user and caregivers Establish and empower service user and caregiver groups Support rehabilitation/livelihood support and social inclusion of service user and caregivers Promote human rights protection Support mobilization and organization of service user/caregiver organizations	Working with the Community Advisory Board (CAB), leaders of social associations, faith based and religious organizations, Health Development Army, Development agents and health extension workers Building capacities (e.g., training) local statutory and non-statutory leaders in service user involvement and empowerment Conducting CAB consultative meetings Awareness raising to the local community about service user and caregiver involvement Strengthening collaboration and involvement of community stakeholders	Improved community awareness about mental health/illness, and service user and caregiver involvement Non-stigmatizing support Improved collaboration of community stakeholders Improved support, inclusion, human rights protections, and rehabilitation/livelihood support of service user and caregivers
Health organization (service provider/managers)	Provide mental health services within primary healthcare Avail adequate and reliable treatment (medication and basic psychosocial care) Ensure competent, compassionate and respectful care services Provide relevant and evidence-based information and treatment choices Ensure services are accountable and responsive Mobilize and organize service user and caregiver groups Encourage social inclusion	Capacity building training about how best to involve and work with service user and caregivers Facilitate involvement to develop and evaluate a plan for service user and caregiver involvement, focused to ensure availability and quality of mental health care	District health office continues to support availability of integrated mental health care in the study site Improved information and treatment choices for accountable and responsive mental health care Improved knowledge of participatory care provision involving service user and caregiver Non-stigmatizing (inclusive) care Availability of competent, compassionate, respectful and caring service providers
Service users	Establish service user groups Advocate for prioritization of mental health service delivery, sustainable medication supplies, and human rights protection for service users Provide peer support, awareness raising about mental illness for the local community Establish collaborations with various stakeholders to empower service users and address unmet social needs	Capacity building training about basics of mental health and illness, understanding service user involvement, mental health related stigma and discrimination, human rights issues and mental health advocacy Facilitation of service user organization and empowerment Facilitation of involvement to develop and evaluatea plan for service user involvement	Improved service user awareness about mental health and mental health services Improved communication between service users, caregivers and service providers Non-stigmatizing attitudes Improved treatment adherence of service users Improved care(pharmacological and basic psychosocial) for service users Improved influence of service user voices and perspectives
Caregiver	Collaborate with service user groups Enhance advocacy for mental health service and protection of service user`s rights Provision of peer support Provision of awareness raising about mental health for the local community Encourage social inclusion Establish collaborations with various stakeholders	Capacity building training about basics of mental health and illness, understanding caregiver involvement, mental health related stigma and discrimination, human rights issues and mental health advocacy Facilitation of caregiver organization and empowerment Facilitation involvement to develop and evaluate the plan for caregiver involvement	Improved caregivers awareness about mental health and mental health services Non-stigmatizing attitudes Improved communication between service user and caregiver Reduced caregiver burden of care, costs Increased economic productivity Improved influence of caregiver voices and perspectives

### Interventions

In the first ToC workshop, participants emphasized three programme levels (service users, caregivers, and district health organisation) where interventions would be necessary. The participants from the second workshop added ‘local community’ as an important additional level. During the meeting with researchers and particularly stakeholder groups, the participants spent much time unpacking the changes and inputs needed from different actors at different layers of the community (e.g., leaders of social organizations, faith-based organizations) and interventions at the community programme level. The final ToC roadmap included four programme levels: i) local community, ii) district health organisation (service providers and health service managers), iii) service users, and iv) caregivers. For each level, various interventions and distinct preconditions (see Table 2) were identified by the participants as necessary steps towards achieving the long-term outcomes.

### Community level

The participants of the ToC workshops reflected on the challenges of the service users and caregivers in the local setting, because they tended to be excluded from social participation in the community, and tended to be of lower socio-economic status or unemployed and at risk of lack secure housing, food, clothing and abuse of their rights. There was no organized community support for service users and caregivers, except the ad hoc contributions of individuals who were supporting some service users on a voluntary basis. The participants identified various barriers (e.g., low awareness about mental illness in the community, lack of service user and caregiver organizations) that needed to be addressed in their local settings. During the two workshops and meetings, the need for community mobilization was prioritized to overcome the socio-economic challenges of service users and caregivers and to empower them to contribute to the strengthening of the mental health system. At the subsequent consultation meetings, several actors, preconditions and interventions were added to the community programme level. For example, participants recommended expanding the composition of the CAB to include more faith-based organizations, traditional healers, social association leaders (e.g., *Idir*; burial societies), HEWs, development agents, and health development army leaders (community health volunteers). The participants emphasised the need to identify and work with these actors as champions at the community level for the following reasons: to facilitate community awareness raising about mental health/illness, to promote roles of service users and caregivers, to help to mobilise community resources, and to expand community-based psychosocial care for mental health. The CAB was assumed to serve as a vital platform for information sharing, community dialogue, strengthening collaborations and involvement of local statutory bodies (e.g., district health office, justice, social and labor affairs) and non–statutory organizations (e.g., social associations, religious and faith-based organization, non-governmental organizations) to organize and support self-help groups (e.g., orphan support group, caregiver organization, service user organization), as well as to overcome the barriers to involvement and facilitate the recovery of service users. HEWs were identified as the key actors for awareness raising activities and stigma reduction in the wider community, due to their links with community leaders, and close relationships to the community and health facility. The recognition of mental health as a key package in the health extension programme was raised in support of the proposal.

The participants made strong verbal commitments to work together, although working plans for specific outcomes still needed to be drawn up (e.g., processes or procedures for awareness raising, packages of training at community level). Participants’ verbal feedback indicated that the ToC process helped to clarify how programme level actors saw their roles and how they could work together. For example, one participant from the stakeholder leaders group explained how the process of ToC development had been helpful:

*‘The discussion was really a learning forum, really forces us to put our thoughts and experiences that we have been doing the last five or so years… discussion helped us critically reflect what we have done so far; the reflection in the process stood out in what we have discussed during the previous ToC workshop; this also pointed to what we are going to work as in a government strategic direction, to strengthen what we have done in PRIME, also showed what we have not done well and where we need to improve particularly strengthening collaboration and CAB….it has been great. *(District official)

As shown on the ToC map and Table 2, at the community level, the participants articulated key preconditions to achieve the intended long-term outcome. In particular, the need for strengthening service user and caregiver associations to function as an important partner in healthcare service improvement was stressed at all levels of the ToC development process. The participants recommended working with the CAB of the PRIME project. During the meeting with stakeholders in Sodo district, priority was given to the need for capacity building training for CAB members and key community leaders to enable them to promote community level awareness raising about mental health/illness, to reduce the impact of stigma and discrimination associated with mental illness and promote the human rights of service users and caregivers. Some participants advised that mental health promotion should be linked to schools.

### Health organization (service providers/managers) level

The workshop participants appreciated the initiative by the government and PRIME to integrate mental health services into primary healthcare, and thus to provide accessible mental healthcare in Sodo district. In order to ensure ongoing sustainability of mental health services, participants identified the following as crucial: to equip healthcare centres with adequate medication supplies, and ensure continued availability of competent, compassionate, respectful and caring service providers. Unreliable medication supplies were raised by both service providers and users as a source of dissatisfaction. During the stakeholder meeting, the officials described several efforts to improve the conditions of service users and practical measures taken since the first ToC workshop (e.g., housing, community-based initiatives to support service users). All the stakeholder groups appreciated these efforts, particularly the commitment of the district health office to improve medication affordability through alternative solutions to cover the costs for those who cannot afford to pay. However, the lack of a consistent supply of psychotropic medications remained a severe problem. The root cause of the problem was a point of heated discussion, with accusations of discrimination against mental health care when compared to physical health service delivery. For example, one health professional currently working in the district administration stated:

*“We have enough money to cover the psychotropic medication, but the problem is unavailability of the medication at stock even sometimes in Emmanuel Hospital…is not in the essential medicines list in their procurement processes. This is lack of attention to mental health and service users within the health systems…human rights violation …we can do or perfectly capable of doing this, but negligence… for example, after PRIME we started doing many things by ourselves.” * (District health official)

The participants discussed the need to advocate and create pressure at federal level about the medication supply problem. The importance of empowering service user associations to advocate for their rights was underlined.

Communication problems between recipients and providers of care, lack of relevant evidence-based information for service users and caregivers about mental health treatment options, including medication types and side effects, were also highlighted as a barrier to better involvement. Staff expressed the need for training about how best to collaborate and involve service users and caregivers within the healthcare system. Several preconditions were articulated, together with necessary interventions (see Table 2) at the health organization level to achieve the intended long-term outcome.

### Service user and caregiver level

The participants voiced their concerns about the lack of service user and caregiver involvement in the mental healthcare system in Sodo district. In the qualitative formative study [54], several barriers to involvement were identified, including severity of mental health condition and lack of a service user and caregiver organization and empowerment. The qualitative study and ToC workshops participants demanded the establishment of an effective service users-caregivers association. The ToC workshop participants listed several tasks that service users-caregivers and their organizations could accomplish to support health system strengthening (e.g., serve as pathways to enhance advocacy for mental health, peer support; see Table 2). The ToC workshop participants debated about the best focus for a new service user and caregiver organization. In the first ToC workshop it was proposed to cover the entire Sodo district, but in the second workshop the participants agreed to start in the capital town (Buie) of the district and gradually scale up because of topographical, logistical and other perceived challenges.

The workshop participants agreed that the district health office needed to take responsibility to facilitate the establishment of representative service user and caregiver organizations, with technical support, e.g. draft charter, to be provided from researchers and professionals in the area of developing policy/charters. The district health office made a public commitment to support establishment of a service user and caregiver association in the district, including the process of certification to become a legal entity. Stakeholders drew on their experience and recommended adopting the model used for HIV/AIDS associations.

At the service users-caregivers programme level, several interventions were identified (See Table 2), including the need for capacity building training for service users-caregivers and to promote the role of non-governmental organizations (e.g. the recently established Mental Service Users Association at Addis Ababa, the Mental Health Society of Ethiopia), and professionals and researchers to empower service user and caregiver for meaningful involvement. The ToC workshops participants also articulated pre-conditions (see Table 2) to be achieved at this level to reach the intended long-term outcome.

### Key assumptions, evidence and indicators

Our ToC identified several assumptions (e.g. willingness of actors to work collaboratively) deemed necessary to be in place for the outcomes to be achieved. The ToC also outlined indicators of success (e.g., comprehensiveness of mental healthcare at primary health, stakeholder satisfaction), as well as the key rationale for the programme along the ToC causal pathway to the intended long-term outcome. A summary of the key assumptions, evidence/experience base and indicators to reach the long-term outcome and intermediate outcomes at the four levels is presented in Table 3.

**Table 3:** Key assumptions, evidence and indicators

Outcomes levels	Assumptions	Evidence/experience base	Indicators
Long-term outcome	Preconditions at the community, health organization, service user and caregiver level met Improved community organizations and inter-sector collaboration	Stated during the workshop by the participants that represented diverse stakeholder groups	Health care system inclusive of service user and caregiver needs(service user and caregiver satisfaction, affordable medication, holistic care, including medication and psychosocial, involvement structure and strategies) Comprehensives and reliability mental healthcare service (pharmacological and basic psychosocial interventions) Established service user and caregiver involvement structures within healthcare systems (e.g. peer support, board/committee members)
Community intermediate outcomes	Willingness of community stakeholders to work collaboratively to mobilize, support and empower service user and caregivers and their organization Willingness of social organizations to promote mental health for their members	Stated during the ToC workshops and meetings by the participants that represented diverse stakeholder groups Research evidence in the local context showed the need [110]	Number of stakeholders involved in ToC workshops Number of stakeholders involved in service user and caregiver support meeting ToC map and narratives Community stakeholders collaborate to empower service user and caregiver groups Number and types of community support generated to empower service users
Health organization	Willingness of health professionals and managers to undergo capacity building training; engage in participatory action research with service user and caregiver’s Commitment to create enabling environment service user and caregiver involvement	Stated during the ToC workshops by the participants Assessed need in the qualitative study Several research findings from HICs [3, 12, 13] and international study [2]	Number of participants attendance at the capacity building training Changes in knowledge and attitudes compared in pre-and-post training Number of members involved in participatory action research activities Reported personal experiences related to ToC model introduction
Service users and caregivers	Willingness by service user and caregiver to undergo capacity building training Willingness to work with health professionals, managers and community Time and resources to engage in involvement activities	Stated during the ToC workshops by the participants Identified needs in the qualitative study Recommended by several studies from HICs [3, 12, 13], LMICs [30, 42, 91, 111] and international study [2]	Number of participants attended capacity building training Changes in knowledge and attitude compared in pre-and-post training Number of participants involved in participatory action research activities Reported personal experiences related to ToC model introduction Service user and caregiver groups organized

## Discussion

In this study, we report about the development of a Theory of Change (ToC) which aimed to integrate service user and caregiver involvement in mental health system strengthening in a primary healthcare setting in a low-income country. The ToC describes service user and caregiver involvement as a complex intervention and makes explicit the hypothesized pathways through which the intervention components interact to achieve the intended long-term goals of (i) improved physical and mental health, work productivity and social inclusion for service users, and (ii) improved life satisfaction and economic capacity for caregivers. The achievement of these long-term outcomes may ultimately lead to improved quality of life and reduced mortality of service users in the Sodo district. The current study drew upon findings from rigorous formative work with key stakeholders and involved diverse representation of service users, researchers and community groups in the co-produced ToC.

The final ToC consists of four programme levels (community, health organization, service user, and caregiver). This is line with the well documented evidence that determinants of mental health and illness are multi-layered [55] and require intervention packages beyond a health facility (e.g., health system, and community levels) [56–58] to address service users’ and caregivers’ multidimensional needs (e.g., health costs, employment, education, housing, social inclusion). Clear articulation and understanding of how different programme levels fit together is essential to guide the coordinated working of multiple actors and agencies and provide a clear picture of what needs to change and how, to achieve sustainable change [59, 60]. For example, district stakeholders identified the availability of rich community resources (also reported in previous study in the district [61]) and multiple actors, particularly at community programme level (e.g., social organizations like Idir) with viable potential to address the psychosocial needs of service users-caregivers locally, with less external support and in a sustainable manner.

In our study, the ToC development process created a forum for knowledge exchange and dialogue among stakeholders about the value of service user involvement, needs, and how to work together to implement this in the local context. The critical reflective discussion with stakeholder groups at Sodo district applied participatory action research (PAR), where participants brainstormed possible solutions to several barriers for effective service user involvement, including the urgency for local solutions (e.g., medication supply challenges, supporting establishment of a service user association). For example, in our separate ToC with professionals (psychiatrists and researchers) and community stakeholders we observed that professionals emphasized long-term outcomes that were more health system focused compared to the community stakeholder groups that tended to emphasize more holistic goals. In the subsequent meeting, the participants discussed this difference of perspective and reached consensus on outcomes that accommodated the perspectives of all participants. Much research evidence indicates that active involvement of stakeholders, including service-users and caregivers, in research ensures that the research evidence is relevant, useful and trusted by all, including the end user (service users) [28, 62, 63]. More importantly, the process created heightened stakeholder buy-in [64], particularly among the political leaders and the community representatives. The ToC process also stimulated collaborative working of stakeholders to empower mental service users-caregivers, which was identified to be starting from a ‘low base’ in the district [47]. ToC approach offers a key contribution to enhance equity and reach of key stakeholders [65], and to incorporate the expertise and knowledge of diverse stakeholder groups` values, needs and preferences. Hence, the ToC development process by itself can be capacity building [66], helping to foster learning and reflection [67], and can facilitate contextualised solutions through a sense of stakeholder ownership of the programme [68–70]. Particularly when ToC employs the techniques of participatory action research, it can serve as a catalyst for learning and promote informed action by supporting stakeholders to achieve sustained positive changes in the local context [35, 40, 71].

In our previous qualitative study [54], several structural barriers (e.g., lack of access to care) were identified that serve to disempower people with lived experience of mental health conditions and undermine involvement within healthcare systems. Recently, the Ethiopian government has made a national level commitment to improve the situation of people with mental health conditions through increasing the availability of mental healthcare services at primary healthcare level [49, 72]. Based on our ToC findings, this initiative needs to ensure good quality of care and interventions to address the high levels of physical, emotional and social suffering and disability [73], excess mortality [74], lack of reliable supplies of medication, and low socio-economic status of service users and caregivers (lack of basic needs, shelter, education) [47, 54]. Many studies from LMICs [75, 76] and high-income countries [3, 12, 13] similarly recommend to address and overcome these structural problems, including opportunities to ensure social inclusion and protection of basic human rights (including healthcare) as required conditions to enable mental health service user involvement and recovery to occur.

In many high-income countries, enabling organisational level conditions (e.g., policy directives, legislation, strategies/guidelines, education and support) are available to support meaningful service user and caregiver involvement in mental health systems [1, 28, 77]. In this regard, the promotion of and effective implementation of national and international instruments for the protection of the human rights of service users; and enacting comprehensive mental health legislation with robust enforcement mechanisms are key areas that need attention in LMICs [26, 78], including Ethiopia. The Ethiopian national constitution [79] clearly guarantees the rights of people with disabilities. The country has ratified the United Nations Convention on the Rights of Persons with Disabilities, which includes both physical and mental disabilities [80]; and employment rights [81]. The Health Policy [82], the revision of the National Mental Health Strategy [72] and the Health Sector Transformation Plan [49] also need provisions to explicitly state how service user and caregivers need to be involved within the mental health system.

In our ToC, we identified strengthening local community collaboration to support service user and caregivers as a major intervention component. Previous studies from Sodo district have indicated that there are potentially rich local resources and capabilities (e.g., diverse community based organizations, community based health workers, assets, micro-finance institutions, social capital, community self-support organisations [47, 61]. These resources are not currently mobilized to support mental health service users and caregivers, and there is still limited inter-sectoral collaboration and community awareness about mental illness [47] that may contribute to marginalisation of service users in society. The community level problems require the participation of a range of societal actors [70]; and the need for community collaboration in the provision of comprehensive community mental health services is well recognized, including in LMICs [83, 84]. The current direction of global mental health care emphasizes strengthening community resources, greater focus to address local priorities and developing local assets to solve local problems [7, 85, 86]. One of the mechanisms of community involvement that has been of high policy interest, and is increasingly supported by research evidence, such as engaging local community stakeholders through Health Committees/Community Advisory Boards (CAB) [87–89]. CABs mediate between communities and health services in many health systems [87] and can be effective to improve the quality and coverage of healthcare, as well as impacting on health outcomes [2, 88, 90].

A healthcare organization programme level was included in our ToC as a key intervention pathway to empower service users and caregivers to play a role to improve the mental healthcare system. A qualitative study of nine service user and caregiver organizations in seven African countries found that one of the important success factors is strategic government level and health organization support that promotes self-determination and service users’ and caregivers’ control over agenda-setting [91]. There are various ways in which healthcare organizations can create an enabling environment for involvement, including development of a culture of acceptance(non-stigmatizing attitudes and eliminating discriminatory practices); allocation of financial, human and material resources, and recognition of experiential knowledge to strengthen the mental health system [3, 12, 13, 92]. At the health facility level, available up-to-date information about service user rights, the nature of their condition, available evidence-based treatment options and services, and provide infrastructure for involvement can enhance meaningful involvement of service users-caregivers [3, 12, 93].

The service user and caregiver programme level was a key intervention pathway in our ToC to mobilize and empower service users and caregivers for mental health system strengthening. Various barriers hinder service user involvement (e.g., severity of mental and physical illness, lack of decision making skills, poor information and insufficient opportunities for choice) [3, 12, 13, 94]. The pervasive stigma operating within the health system, the local community and individuals, and limited empowerment and mobilization of service users can have disempowering effects on active involvement [54]. Moreover, resource limitations, practical and logistical challenges are important barriers to mental health service user involvement in LMICs which must be addressed [95]. The importance of empowerment of service users and caregivers (at individual and group level) has been recognized widely in mental health system strengthening [12, 13, 94]. At the individual level, empowerment involves addressing both knowledge (e.g. training about effective communication, advocacy, working collaboratively) [12, 13, 31, 96] and health challenges (e.g., medication supply) [12, 13]. Training can improve self-esteem and self-advocacy, assertiveness, confidence and hopefulness of service users and promotes recovery [12, 13, 96]. At the group level, empowerment can be achieved through organization of service users-caregivers.

Several studies identified a range of benefits of service user and caregiver organizations to service users and caregivers, health professionals and health systems. Empowering service users to self-organize and advocate for their interests can promote their recognition of a sense of belonging, and develop their confidence, strengths, resources and skills [26, 91]. This also ensures a collective voice to influence and lobby for policy and legislative reforms [91, 94, 97]. Service user and caregiver associations operate at different levels to protect the rights of service users [76, 91, 98] and there are reports that participation in self-help groups is an independent predictor of improved functioning in areas like voting, marriage, attending festivals, treatment adherence [99], fostering greater acceptance of service users by their family members and by the community, consistent treatment and better outcomes [100]; income generation and quality of life [76]. Self-help groups are important platforms for exchange of individual experiences and mutual support that can lead to better quality of life and insight about how best to cope with their situations [91]. However, there are few service user and caregiver associations in LMICs; for example, in Ethiopia The Mental Service User Association was established in Addis Ababa in 2018, and there is only one association of caregivers at the national level [47].

The ToC workshop and qualitative study participants [54] underlined the importance of capacity building training for service providers/managers and service users-caregivers to enable service user and caregiver involvement. Our finding aligns with Carman et al. [31] who identified the importance of technical support, strong leadership, preparation of service users, health professional and other stakeholders, and partnerships as key facilitators in implementation of multilevel frameworks for service user and caregivers involvement. Various studies also recommend to address the knowledge, attitudes and skills needed for service providers, health administrators at all levels of the healthcare system and service user and caregivers to implement models of service user and caregiver involvement [3, 12, 13, 96]. Participatory action research oriented approaches, such as photovoice, can create longer and repeated opportunities for social contact between service user and caregivers and care providers [101–103] and lead to attitudinal change. Photovoice is a multistep participatory action research methodology whereby service users are equipped to represent their stories, including their perceived health and work reality, using photographs, with the goal of impacting an aspect of the system and/or policy [104–106].

### Strengths and limitations

The participatory ToC development and refinement process brought together diverse stakeholder groups including service users and caregivers to work collaboratively to strengthen service user and caregiver involvement in mental health system, which is a novel approach in LMICs. Their involvement in the ToC development created a sense of ownership and stakeholder buy-in, which is important from the point of view of follow-through on implementation. The overarching goal of this study, was to develop active service user and caregiver involvement in mental health system strengthening. We did not attempt to stratify the ToC process as a mechanism to overcome power imbalance among the multi-stakeholder groups; because the process of bringing diverse stakeholder groups through facilitated interaction can offer better social contact opportunities. Such an approach has been recommended as an effective mechanism to improve attitudes and reduce stigma [107, 108]. Nonetheless, we drew upon the rich experience of the facilitators and their awareness of the potential power difference to intentionally encourage service users, caregiver and other less vocal participants to engage more. The principal facilitator (AF) started the process with a simple and concrete example to explain about ToC to make it understandable for all participants, and to ensure the process achieved its intended goal with the highest possible buy-in from the participants.

Despite this, even with encouragement from the facilitator, the service user participants were not as active as other participants in voicing their views during the ToC workshop. Similar observations have been made previously when developing a participatory mental health care plan in a neighbouring rural Ethiopian district [109]. We were able to draw upon findings from in-depth interviews conducted in the formative phase, which may have helped to mitigate this problem [54]. We don’t think there is a ‘one-size-fits all’ recommendation to make the ToC process more inclusive and overcome the inherent power differentials that could exist in multi-stakeholder discussions. However, we believe that, in addition to the key role of trained and experienced facilitators` efforts to make the process more inclusive, it is helpful to train/equip the participants to engage in discussions and to provide clear initial orientation about the ToC process using local metaphors.

The ToC is mainly based on the views of stakeholders from a limited geographical area of Ethiopia (Sodo district) and may not be transferable to other settings.

## Conclusions

The development of ToC and the involvement of diverse community representatives in the process was critical in terms of understanding the context of the programme intervention, to identify components of interventions, and articulate preconditions and underlying assumptions. The participatory approach, systematically applied, gives structure to the identification and articulation of programme theory, an important step of service user involvement in mental health systems strengthening initiatives. This study adds to the limited empirical data on best practices to develop service user and caregiver involvement, particularly in LMICs. The next step will be to pilot and evaluate the model using participatory action research methodology in this rural Ethiopian setting.

## Data Availability

Not applicable.

## Abbreviations

CAB: Community Advisory Board
FMOH: Federal Ministry of Health
HEWs: Health extension workers
HIC: High income countries
HSTP: Health Sector Transformation Plan
LMICs: Low- and middle-income countries
Emerald: Emerging mental health systems in low- and middle-income countries
PRIME: PRogramme for Improving Mental health carE
ToC: Theory of Change
WHO: World Health Organization
